# Periodization Theory: Confronting an Inconvenient Truth

**DOI:** 10.1007/s40279-017-0823-y

**Published:** 2017-11-30

**Authors:** John Kiely

**Affiliations:** Institute of Coaching and Performance, School of Sport and Wellbeing, University of Central Lancashire, Preston, PR1 2HE UK

## Abstract

Periodization theory has, over the past seven decades, emerged as the preeminent training planning paradigm. The philosophical underpinnings of periodization theory can be traced back to the integration of diverse shaping influences, whereby coaching beliefs and traditions were blended with historically available scientific insights and contextualized against pervading social planning models. Since then, many dimensions of elite preparation have evolved significantly, as driven by a combination of coaching innovations and science-led advances in training theory, techniques, and technologies. These advances have been incorporated into the fabric of the pre-existing periodization planning framework, yet the philosophical assumptions underpinning periodization remain largely unchallenged and unchanged. One particularly influential academic sphere of study, the science of stress, particularly the work of Hans Selye, is repeatedly cited by theorists as a central pillar upon which periodization theory is founded. A fundamental assumption emanating from the early stress research is that physical stress is primarily a biologically mediated phenomenon: a presumption translated to athletic performance contexts as evidence that mechanical training stress directly regulates the magnitude of subsequent ‘fitness’ adaptations. Interestingly, however, since periodization theory first emerged, the science of stress has evolved extensively from its historical roots. This raises a fundamental question: if the original scientific platform upon which periodization theory was founded has disintegrated, should we critically re-evaluate conventional perspectives through an updated conceptual lens? Realigning periodization philosophy with contemporary stress theory thus presents us with an opportunity to recalibrate training planning models with both contemporary scientific insight and progressive coaching practice.

## Key Points

**Table:** 

The science of periodization has, for the past seven decades, borrowed substantially from the science of stress to substantiate certain fundamental periodization principles. Yet although stress science has dramatically diverged from its historical roots, periodization theory continually recycles old stress dogma as justification for contemporary doctrine.
Fitness adaptations, subsequent to imposed training stressors, are greatly influenced by the neuro- and bio-chemical backdrop upon which training stimuli are overlaid. This neurobiological context is, in turn, greatly influenced by background levels of psycho-emotional stress and the set of emotional expectations and interpretations associated with the imposed training challenge.
The phenomenon of path dependence provides a lens through which to contextualize how the legacy of prior beliefs exerts a constraining influence on current practice, thereby suppressing conceptual clarity and coaching creativity.

## Author’s Note

Every year, the *Edge.org* poses a single question to a collection of scientists, technologists, and social influencers. In 2011, that question, proposed by Harvard’s Steven Pinker, was “*What scientific concept would improve everybody’s cognitive toolkit?*” Among the responses, one from Columbia’s John McWorther stands out as particularly thought provoking in the context of current theories of athletic preparation. McWorther’s suggestion, the phenomenon of *path dependence*, captures the notion that often “*something that seems normal today began with a choice that made sense at a particular time in the past, and survived despite the eclipse of the justification for that choice*” [1].

The paradigmatic example is the QWERTY keyboard. Historically, the QWERTY interface reduced the frequency of mechanical jamming by separating the keys of the most commonly used letters. Although technological advances eradicated this risk decades ago, the legacy of the solution, to that now non-existent problem, persists. In 2008, the Nobel Prize in Economics was awarded to Paul Krugman for a body of work illustrating the hidden path-dependent influences shaping industrial trade patterns. Krugman, amongst others, suggests path-dependent phenomena are pervasive in life. Operating not only within socio-industrial settings but whenever prior solutions become enshrined in practice and are routinely perpetuated, despite a change in the underlying circumstances from which those solutions arose. Put plainly, path dependence emphasizes that where we go next depends not only on where we are now, but also where we have been [2].

As a relevant example within sports science and medical domains, consider the long-standing belief that adolescent weight training compromises skeletal health. How did this belief arise? Although difficult to definitively trace, a preeminent researcher in this field, Avery Faigenbaum, suggests the *strength training stunts your growth* myth arose from a 1960s report claiming that children performing heavy manual labor were short in stature [3]. These children lived in a mountainous region of post-war Japan, and worked several hours a day under chronically compromised nutritional conditions. Nevertheless, despite these obvious confounds, an overly simplistic conclusion seeped into our collective consciousness and fossilized into a self-perpetuating pillar of belief. Eventually, although the origin story was forgotten, the belief persisted and remained remarkably culturally resilient despite decades of disconfirming evidence.

Path dependence reminds us that the philosophical bedrock of many inherited doctrinal beliefs often remain shielded from skeptical scrutiny, sheltered by an ideological inertia. Sometimes, consequently, re-evaluating embedded belief systems requires we excavate the deep-seated often-forgotten foundations upon which traditional assumptions are supported.

## Introduction

Few dimensions of elite sports performance are as important, as complex, as experimentally impenetrable, and as shrouded in historical myth as the topic of training planning: the periodization of training. Many periodization approaches exist, each offering differing rationales and templates for the sub-division of the program into sequential, specifically focused training periods designed to prepare athletes for peak performance during prioritized time frames.

The late Mel Siff once described periodization as an exercise in stress management [4]. In fact, since periodization’s first formulation, concepts borrowed from the science of stress have been persistently offered within coaching and academic literatures as justifications for pivotal theoretical assumptions. In recent decades, however, the science of stress has evolved far beyond its historical roots. Yet despite this evolution, certain long-standing stress precepts remain firmly embedded within contemporary periodization culture. Thus, although the foundations upon which periodization logic was supported have shifted substantially, culturally we continue to re-cycle prior interpretations of archaic stress theory to justify current planning practice. From this perspective, periodization’s historical foundations appear rooted, in a path-dependent manner, in an outdated science. Accordingly, the re-calibration of pivotal periodization assumptions, with current theoretical insights, may reveal new insights illuminating future training planning innovations.

## A Brief History of Stress

The evolution of the science of stress began in earnest in the first decades of the twentieth century. Famously, in the 1920s, Harvard’s Walter Cannon—echoing Bernard’s earlier concept of a balanced *milieu interieur*—suggested arousal shifted an animal’s set of internal steady-state conditions, which he termed *homeostasis*, away from stable habituated set-points [5]. This disequilibrium, in turn, stimulated catecholamine secretion, specifically adrenaline, thereby powering the ‘fight or flight’ emergency response designed to alleviate the imposed challenge, quell the biological disturbance, and facilitate a return to homeostatic normality [6].

A decade later, Hans Selye, switching attention from the catecholamines of the adrenal medulla to the glucocorticoids of the adrenal cortex, began the body of work destined to revolutionize the field. During his early career, Selye observed that rodents who experienced diverse physiological discomforts displayed surprisingly similar stereotypical responses. Regardless of whether rats were electrically shocked, fatigued, starved, or exposed to temperature extremes, observed maladaptations shared a common non-specific trajectory. In his landmark 1936 letter to *Nature*, Selye described a triad of symptoms, adrenal enlargement, gastrointestinal ulceration, and atrophy of the thymus, which he claimed were predictably elicited by multiple biological insults [7].

The apparent universality of this pathological triad prompted Selye’s formulation of the general adaptation syndrome (GAS). The GAS encapsulated Selye’s core thesis that all biological challenges were countered in a predictable fashion, progressing through the same sequential phases: first alarm, then resistance, and, if the challenge was overwhelming, resulting in the same end product, exhaustion. Selye deployed an engineering term to describe the animal’s response to such perturbation, redefining *stress* as the “*non*-*specific response of the body to any demand made upon it*”, and *stressor* as any *noxious* agent stimulating the GAS response [8].

As the twentieth century entered its final quarter, our understanding of stress and its associated vocabulary—homeostasis, *fight or flight*, the adrenal master gland, GAS—was shaped by these early pioneers. Although superficially recognizing that we each have individually distinct thresholds, set-points, strengths, and vulnerabilities, Selye envisioned the stress response as a stereotypical species-wide phenomenon. The implicit sub-text was of an assumed conformity to imposed demands, whereby stress-induced adaptive responses were tightly bound around the predictable trajectory of the GAS response.

### Impacting the Coaching World

Selye once remarked that he never considered the application of his research to sporting domains [9]. Nevertheless, astute coaches quickly recognized its relevance [9, 10]. Influential early translators of Selye’s work to sporting contexts included innovative Australian swim coach Forbes Carlisle, in 1955, track and fields Fred Wilt in the early 1960s, followed by swimming’s legendary James ‘Doc’ Counsilman in 1968 [9–11].

Today, Canon and Selye’s legacies remain enshrined within the science of periodization, as evidenced by the persistent citing of homeostasis and GAS as theoretical platforms upon which contemporary planning theory is founded [12–14]. The world’s largest strength and conditioning certification body, the National Strength and Conditioning Association, for example, notes the importance of GAS and homeostatic principles within that organization’s publications, stating: “*GAS is one of the foundational theories from which the concept of periodization of training was developed*” [15]. Similarly, within the academic literature, the only periodization reviews published in high-ranking peer-reviewed journals to date both cite Canon and Selye, noting, for example, that the biological background of periodized designs exploits homeostatic regulation and stress adaptation as fundamental theories of human adaptation [16, 17].

### Confusion and Controversy

In the immediate post-war era, Selye’s teachings dominated academic and popular understanding of the stress phenomenon. Concurrently, however, a more psychology influenced research tradition was beginning to navigate its own evolutionary arc. As the century progressed, and these paths transected, ideological conflicts inevitably emerged [18].

Homeostasis and GAS were both firmly biologically entrenched concepts, an issue Selye acknowledged late in life, noting he had long envisioned stress as “*a purely physiological and medical phenomenon*” [19]. In contrast, psychologists interpreted the stress response as primarily a cognitive event, emerging directly from “*a mismatch between individuals’ perceptions of the demands of the task, and their perceptions of their resources for coping with them*” [20].

Central to these debates was the origin of the unidentified signal responsible for initially triggering the alarm response, the so-called *first mediator*. Selye predicted, and fruitlessly searched for, a biological first mediator. More psychologically oriented researchers, however, argued the first mediator was psycho-emotional in genesis, in essence suggesting that events stimulate a stress response only when appraised as ‘threatening’ [18, 21, 22].

Perhaps most notably, throughout the 1960s and 1970s, John Mason—working within Joseph V. Brady’s ground-breaking inter-disciplinary group at Walter Reed Memorial—demonstrated that the stress response varied substantially as a function of the situation, the individual, and the individual’s history. Mason’s work highlighted, for example, that when the *noxious psychological concomitants* of physical stress were reduced or removed, the GAS either dissipated or disappeared [18, 23]. Simultaneously, classic Selye-inspired theory was straining to accommodate evidence demonstrating that neither homeostasis nor the stress response was static, but varied dynamically under the influence of life history and oscillating biological rhythms. Conventional theory, as illustration, could not eloquently explain why blood pressure fluctuates markedly throughout the day and often remains elevated long after stressors are removed [24].

As the twentieth century entered its final quarter, the explanatory limitations of Selye’s paradigm were increasingly exposed. Most notably, the portrayal of stress as a predictable biologically mediated phenomenon was undermined by (1) the demonstrable effects of non-physical factors on physiological stress responses, and (2) increasingly convincing evidence that stress responses were not generalized and non-specific, but highly individualized and context specific [25].

### Revolution to Evolution

As the unifying explanatory power of Selye’s paradigm eroded, the field fragmented. Into this conceptual vacuum, various theories were proposed, but without achieving widespread acceptance [26]. Such was the state of the field when Sterling and Eyer (1988), embracing multi-disciplinary insights, proposed the concept of *allostasis* [27]. Allostasis suggests that organisms maintain physiological stability by anticipating ‘needs’ before they arise, and by mobilizing a diverse breadth of neurological, biological, and immunological accommodations to counter these emerging challenges [26, 28, 29]. To facilitate this prediction, multi-source information streams are blended with expectations and prior experiences to estimate the ‘threat’ posed by upcoming challenges. Subsequent to this prediction, multiple preemptive remediating actions, calibrated to that perceived threat, are reflexively launched to protect current and future function, thereby promoting survivability.

Allostasis, accordingly, is not a specific set of tightly controlled homeostatic conditions that must be defended, but a set of collaborative processes that strategically deploy resources to preserve functionality in an unpredictable and dynamically changing environment. Consequently, and in contrast to Selye’s model, allostasis recognizes that the neurobiological imperative is not to seek homeostatic permanency (‘*stability through constancy*’), but to sensitively pre-empt and respond to emerging challenges by orchestrating multi-level system-wide coordinated compensations (‘*stability through change*’) [24, 28].

#### Allostatic Accommodation and Load

When the allostatic state is perturbed, a broad sweep of neurological and biological sub-systems collaboratively co-modulate outputs to accommodate imposed demands. Drastic or persistent *allostatic accommodations*, however, impose a burden: an *allostatic load* [29]. When operating efficiently, well-calibrated allostatic accommodations sensitively emerge in response to current and anticipated perturbations. These accommodations facilitate positive adaptation for minimal accruing allostatic load, and enhance resilience to future similar stress exposures. In contrast, when allostatic responses are inadequate, overwhelmed, or persistently activated, then excessive accommodative shifts drive accumulating allostatic load [28–30].

Although the burden of accumulated load can be gradually alleviated, the legacies of repetitive cycles of accommodation persist as residual traces of neuro-plastic *wear and tear*. Inevitably, the progressive accumulation of these plastically embedded residues impose penalties. Accordingly persistent or excessive allostatic accommodation drives accumulating load, thereby escalating wear and tear and eroding resilience to future allostatic impositions. This progressive neurobiological wear and tear ultimately manifests as some blend of psycho-emotional, physiological, neurological, immunological, and/or behavioral impairment [30].

Thus, allostatic theory suggests that, when challenged, the organism does not reflexively mount a biologically mediated GAS response powered via the actions of lone families of chemical messengers—Canon’s catecholamines; Selye’s glucocorticoids—as it strives to regain a notionally optimal set of steady-state conditions. Instead, entangled networks of neural and biological collaborators orchestrate concerted responses, deploying arrays of systemic mediators modulated through densely inter-connected non-linear feedback and feedforward linkages [29–31]. Allostasis, accordingly, is the complex set of integrated emotional, physiological, immunological, and psychological processes that intimately collaborate to establish a new set of internal conditions best fitting current circumstances [26]. Through these agile adaptive mechanisms, functional robustness on a macro-scale is preserved by persistent synergistic co-modulation on a micro-scale. A phenomenon previously eloquently described as “*the beautiful paradox of seeming constancy, despite continuous change*” [32].

#### The Brain as a Master Gland

Selye envisioned biological stress as largely independent of the brain. Allostasis, in contrast, firmly positions the brain as the master organ responsible for orchestrating all central and peripheral responses to imposed challenges [27, 33]. The rapid evolution of neuroimaging techniques has recently validated this assertion. Importantly, contemporary investigations demonstrate that it is the core emotional regions of the brain—highly evolved sites within the amygdala and basal ganglia—that are the first to register challenge, mediate accommodative responses, and are the first networks to exhibit neuro-plastic wear and tear subsequent to unalleviated load [30, 34].

Collectively, these mid-brain modules function as densely interconnected processing hubs, serving to integrate cognition, descending from higher cortical regions, with sensory information emanating from peripheral and visual centers. Such insights affirm that when a perceived change in circumstances alters sensory input, this change is evaluated by the emotion-processing circuitry (along a continuum ranging from ‘benign to threatening’) and an emotional resonance attached to the event. This emotional evaluation subsequently adjusts circulating levels of neurotransmitters, neuromodulators, neuro-hormones, and neural growth factors. These localized neurochemical changes subsequently customize the cascade of downstream biochemical and hormonal responses mobilized to cope with the anticipated challenge [30, 33, 35]. In essence, emotion calibrates the chemistry of the stress response to perceived context.

Contemporary findings thus illustrate that the long sought-after *first mediator* is not a biological event, but a change in emotional resonance driven by interpretation of sensory events and/or cognitive circumstances [30]. This emotional evaluation subsequently amplifies or dampens the sensations and perceptions deemed *immediately* pertinent to survival, thereby modulating behaviors and motivational drives. Crucially, these emotionally induced neurochemical alterations are not directly dictated by the intensities of imposed stimuli, but by the emotional resonance afforded the stress-inducing event [28, 30, 33]. Consequently, even when stressors seem far removed from emotional significance, such as cold exposure or laboratory-induced histamine reactions, biological responses can be readily modulated and healing times dramatically extended or foreshortened, simply by manipulating the emotional context [36–38]. From this perspective, the stress response is—at its most fundamentally irreducible level—a system-wide survival-promoting neurobiological preparation to cope with anticipated threat, driven by emotional evaluation.

**Fig. 1:**
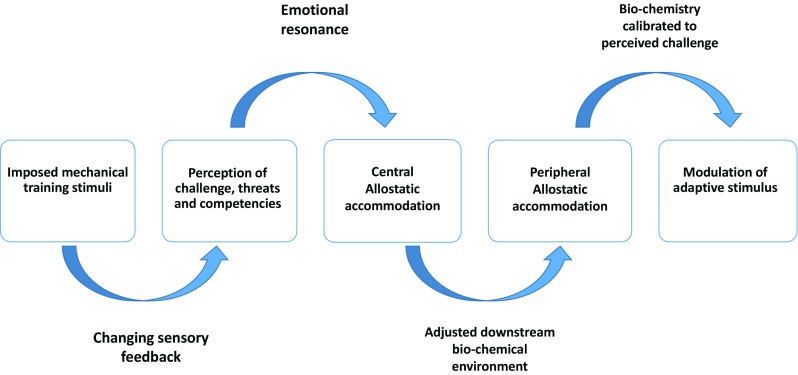
Translation of mechanical stimuli to adaptive response

Specifically, in relation to training planning theory, unquestionably, the mechanical and energetic challenges imposed by physical training are the primary instigators of the sequence of neural and biological events that subsequently drive fitness adaptations. Crucially, however, this contemporary updating of Selye’s stress paradigm reveals that the set of adaptations launched in response to training are strongly and inextricably entwined with, and modulated by, background psycho-emotional influences (see Fig. 1).

## Stress and Athletic Outcomes: The Evidence

### Stress and Injury

Existing evidence supports a firm association between general life stress and sports-related injury. A recent meta-analysis, for example, concluded that a prior history of elevated psycho-emotional stress was a strong predictor of injury occurrence, and demonstrated significant relationships between stress-predisposing personality traits and negative training outcomes [39]. Similarly, the stress-accentuating traits of ‘self-blame’ and ‘perfectionism’ significantly contributed to an increasing injury probability [40, 41]; as did the corrosive influence of accumulating ‘daily hassles’ [39, 42] and periods of high academic stress during the elite college football season [43]. Likewise, athletes with elevated pre-season anxiety were more likely to be injured than their less anxious peers [44] and, following athletic injury, increased psycho-emotional stress diminished the effectiveness of return-to-play processes [45, 46].

### Stress and Performance

Within medical contexts, an extensive literature demonstrates that excessive life stress negatively influences health outcomes [47]. Within athletic preparation contexts, a growing evidence base illustrates the detrimental consequences of stress history, elevated life stress and/or personal predispositions to high stress reactivity on training and performance outcomes [40, 42, 45]. As example: recent research demonstrates that low stress resilience compromises cardiovascular and maximal power training adaptations [48]; high levels of self-rated psycho-emotional stress diminishes positive fitness adaptations following highly controlled training interventions [43, 48]; and elevated psycho-emotional stress compromises training outcomes in well-conditioned triathletes [49]. Furthermore, running economy remained impaired for sustained periods following significantly stressful life events [50], and heightened stress impeded training gains and muscular recovery following resistance exercise [51, 52].

In summary, mounting evidence illustrates that excessively accumulating multi-source stress variously downregulates the immune system, motor coordination, cognition, mood, metabolism, and hormonal health; thereby dampening positive adaptation, diminishing athletic performance, elevating injury risk, and compromising recovery and recuperation [41]. Consequently, athletic populations exposed to excessive stress and/or those constitutionally pre-disposed to high stress reactivity appear particularly vulnerable to the extended family of stress-related syndromes typified by overtraining, underperformance, overuse, burnout, chronic fatigue, immunosuppression, and depression-like symptoms.

### Psycho-Emotional State as a Training Variable

As research documenting the negative consequences of chronically elevated stress grows, so too does evidence demonstrating the impact of acute emotional manipulation on training outcomes. As illustration: visually manipulated psycho-emotional states altered hormonal levels and subsequent strength-training outcomes in highly-trained male individuals [53]; imposing an additional pre-training emotional load increased perceived exertion and diminished physical performance in competitive athletes [54–56]; and heightened anxiety impeded the accuracy of sports-related skills [57].

More positively, the health-promoting benefits of stress-alleviating interventions are overwhelmingly supported within the medical literature [58]. More specifically in relation to training adaptation and injury-related contexts, evidence continues to grow. Recent investigations, for example, demonstrate that prevention strategies moderating psycho-emotional stress successfully reduced injury rates [39], and support the conjecture that positive expectations enhance training outcomes [59]. Furthermore, emotional regulation interventions reduced the negative consequences of stress accumulation in long-distance runners, and have subsequently been suggested as logically enhancing cardiovascular adaptations following endurance training [60, 61].

## Applied Implications: Designing a New Planning Reality

The periodization paradigm is built on the implicit assumption that mechanical loading parameters directly dictate biological training adaptations. Periodization teachings continually reinforce this assertion, as reflected in recent statements suggesting, for example, that “*the overall homeostatic stress of an exercise bout is determined by the interaction of factors such as exercise intensity and duration”* [62]. This perspective unquestionably contains a superficial ‘truth’, yet remains incomplete. Mechanical training stressors do serve as the primary stimulus for, yet are not the sole drivers of, fitness adaptations. Instead, imposed training stressors percolate through a sequence of complex interacting modifying filters before eventually manifesting as fitness responses. Some of these filters, genetic inheritance, training histories, and nutritional states, are widely appreciated. The rationale and evidence presented here, however, suggests a further layer of less fully acknowledged psycho-emotional considerations which, although non-biological in origin, significantly influence biological training adaptations.

**Fig. 2:**
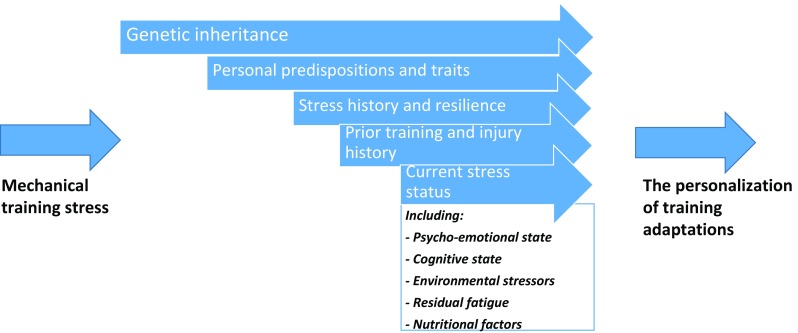
Biological and non-biological filters personalizing the training-induced stress response

Collectively, these modulatory influences interact to shape a uniquely personalized adaptive terrain, upon which mechanical training stressors are overlaid (see Fig. 1). This multi-dimensional adaptive landscape ensures that training responses are deeply customized to the individual, their traits, history, and current neurophysiological and psycho-emotional contexts (see Fig. 2). The highly individualized nature of training adaptation is reflected in apparently contradictory findings illustrating that:

1. When fitness responses are analyzed at an inter-individual level, participants engaging in similarly structured programs typically exhibit widely varying training adaptations [63–65]
2. Yet when averaged group-based adaptations are compared following differently structured training programs, inter-group responses typically remain equivalent [66–68].

These superficially paradoxical findings make sense only when contextualized against the extensive, multi-dimensional inter-individual adaptive variability evident when collections of humans engage in physical exercise. This rationalization highlights the futility of arguments, consuming much of periodizations published history, whereby proponents of specific periodization templates claim superiority over other theorists planning models [69–71]. The claim that a universally ‘best’ periodization framework exists, however, is only sustainable if humans respond to imposed training stress along predictable trajectories, in generalized timeframes, and conforming to predictable dose/response relationships. In the past, Selye’s theories were cited to support such conjecture. Contemporary evidence, however, clearly demonstrates this position is no longer logically defensible.

### Stress, Emotion, and the Measurement Problem

Psycho-emotional stress is an inherently nebulous phenomenon arising subsequent to the integration of neural and biological outputs merging under the influence of genetic, perceptual, experiential, and situational factors. As with many versatile terms used indiscriminately in everyday and scientific conversations, there is no single universally accepted definition of ‘stress’. The problem is not that the term has no clear meaning, but that it has different meanings in different contexts [72]. This definitional ambiguity, in tandem with the complex neurobiology underpinning the stress phenomenon, ensures no single ‘gold standard’ measure of stress exists [73].

However, many subjective assessments commonly used within sporting contexts, such as formal questionnaires and/or self-rating metrics, do reflect facets of psycho-emotional state, thereby providing partial snapshots of experienced stress. Similarly, as autonomic nervous system activity is a major regulator of emotional state, heart rate variability—an objective estimation of autonomic nervous system function—provides a biologically oriented indicator of current stress conditions [74]. It is also worth considering information emanating from more informal processes, such as an experienced coach’s evaluation based on behavioral observations and coach-athlete dialogue. Although each of these data streams is inevitably flawed, each captures a differently focused fragment of pertinent information. Consequently objective, subjective, and experiential-led evaluations provide a varied menu of assessment options, which may be flexibly customized to best fit the situational-specific constraints of any coaching context.

#### Distinguishing between Information and Insight

The proliferation of newly emerging assessment technologies undoubtedly holds the potential to inform planning practice, yet also presents distractions and challenges. Key amongst these challenges is our natural tendency to prioritize readily empiricized metrics (such as weights, times, heart rates, speeds, and distances), at the cost of de-emphasizing parameters that are not easily quantified (such as psycho-emotional state, cognitive load, belief, and expectation). Ultimately, as framed in the famous quote commonly attributed to business theorist Peter Drucker, “what gets measured, gets managed” [75]. Measurability, however, does not directly reflect importance. The subsequent danger is that we disproportionately bias training theory towards ‘managing’ readily measureable physical dimensions of training, and unduly neglect empirically impenetrable psycho-emotional considerations. The most obvious remedy for such measurement-induced myopia is the clarity bestowed by a conceptual model that, informed by contemporary scientific insight, is optimally aligned with objective reality.

### Reframing the Performance Planning Problem

Crucially, and contrary to the message perpetuated within periodization theory, the sweep of evidence presented here implies the worth of the training plan is inseparably entwined with the athlete’s set of perceptions, expectations, associations, doubts, concerns, and confidences implicitly bound to that plan. These psycho-emotional considerations, while ignored by the periodization literature, directly influence physical training adaptations. Despite this conventional oversight, some guidelines for practice already exist within the broader sports science literature.

Such insights suggest that we should, for example, progressively nurture an athlete’s understanding of the training plan, belief in the plan, ‘buy-in’ to the plan, and athletes ‘sense of purpose’, ‘sense of ownership’, and ‘sense of control’ associated with the plan [76]. Similarly, we should install formal and informal feedback processes, thereby providing athletes with a non-confrontational means to voice opinions, doubts, and grievances; we should ensure effective athlete-coach feedback and feed forward communications flow, thereby reducing ambiguity and uncertainty; and  we should educate coaches on the potential stress-amplifying influence of their personal leadership and management styles [77, 78]. Furthermore, we should nurture supportive training processes, training-group cultures, and team dynamics [79], and we should integrate strategies to positively influence mood, perceptions, mindsets, attitudes, risk appraisal, anxiety, trust, coping skills, and interpretations of challenge into the training program [55–57].

Acknowledging that emotional backdrop is a key regulator of training adaptation also highlights the possible benefits of integrating pre-training interventions and routines—designed to accurately calibrate the emotional state with desired session objectives—into habitual training processes. Crucially, just as we target physical capacities with a progressive training plan, we can similarly seek to promote the athletic skills of emotional robustness and stress resilience by programming challenges progressively strengthening these capacities [80]. Such philosophical reframing emphasizes that effective training planning demands more than simply empirically forecasting future mechanical loading parameters. Consequently, our vision of effective training planning should be broadened, beyond purely the mechanical prescription of future training parameters, to embrace this new reality.

### Recalibrating Theory and Practice: So What Can We Do?

**Fig. 3:**
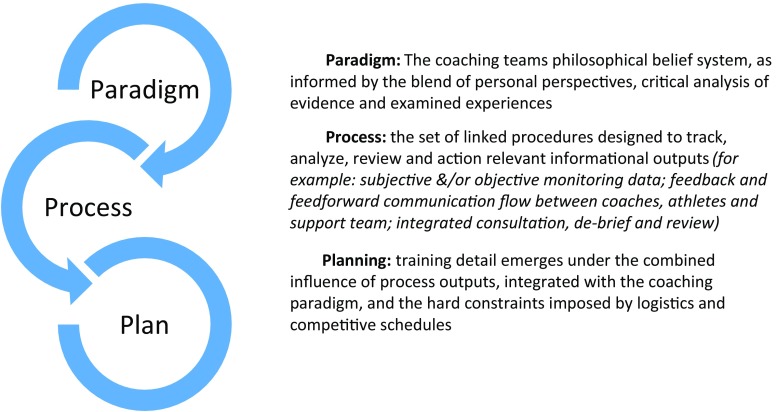
Planning detail as an emergent property of process design

Logically, a broad planning framework should be outlined and starting points, checkpoints, and endpoints agreed. However, within this sparse planning skeleton, training evolution may be most productively driven by emerging time-sensitive ‘information’, captured by well-crafted process ‘outputs’. Such processes may be subjective and/or objective; low or high tech; regular or occasional, and involve varying levels of athlete/squad contributions. The design of such processes sensibly depends on situation-specific variables such as: coaching philosophy, coach/athlete(s) beliefs and preferences, performance needs analysis, experience and training-specific education of the athlete(s), logistical limitations, resource constraints, communication frequency, appropriate application of available technologies and the hard constraints imposed by competitive schedules (see Fig. 3).

Beyond these broad rubrics, however, we have no empirically validated rules, and few specific guidelines. Instead, we are faced with a series of complex trade-offs and negotiations. We need, for example, to navigate between planning rigidity, on one hand, and a formless lack of training direction, on the other. We need a structured training framework, yet one that is flexible and tolerant of change. We need goal-directed coherence, but simultaneously must facilitate seamlessly consistent course corrections in response to dynamically emerging information. Insufficient variation (training monotony) amplifies the probability of negative outcomes, yet too much variation disperses adaptive energy and dilutes training gains [69]. Persistent change drives positive adaptation, but sudden change elevates injury risk [81]. We need a focus on event-specific movement skills, but excessive specificity accentuates structural wear and tear and amplifies the probability of overuse syndromes [82]. Effort must be balanced with recovery. Desired benefits must be weighed against inevitable risks.

Despite periodization theory’s implicit assumption that there is a *one best way*, contemporary evidence compellingly illustrates that there are no generalized formulaic solutions to these planning puzzles. We could nevertheless argue that traditional periodization theory is a benign influence, and that periodization principles should only be interpreted as generalized, but helpful, guidelines. The counter-point, however, is that periodization philosophy perpetuates a belief system founded on twin falsehoods (both tracing back directly to interpretations of Selye’s seminal work): firstly, the supposition that adaptation to physical exercise follows a generically predictable trajectory and, secondly, that biological training outcomes are directly mediated by physical training parameters.

#### Allure of Convention and the Benefit of Doubt

The confusing paradox of human cognition is that we make our best decisions not when we confidently revert to automated rule-based assumptions, but when we are uncomfortably aware of the novelty inherent in every complex situation [82]. If we uncritically disseminate periodization assumptions, because of some misplaced loyalty to tradition, then we are willfully perpetuating a misplaced confidence in a distorted reality. Certainly, there appears little wrong with employing any particular periodization template. It is imperative, however, that we are mindful of the logical errors, oversights, and misconceptions implicit in periodization’s philosophical underpinnings. This skeptical awareness is an essential first defense against the decision-making complacency arising when we are lulled into a false sense of security by the persuasive comfort of convention and the appealing, yet illusory, scientific legitimacy of periodization philosophy.

## Conclusions: A Refined Vision for a New Reality

The rationale underpinning the periodization paradigm was eminently sensible when contextualized against the cultural and scientific landscape of the early to mid-twentieth century. A landscape dominated by the linear logic of Newtonian physics, Descartes *man as machine* metaphor, and the regimented modular planning approach advocated by Frederick Winslow Taylor’s *scientific management* doctrine [69]. Selye’s depiction of the GAS as a generic predictable biological response to imposed mechanical stress dovetailed seamlessly with this philosophical world view. Subsequently, in our cultural eagerness to formulate and justify a comprehensive planning model, it appears we sympathetically over-interpreted a limited evidence base through this flawed philosophical filter.

When contextualized through the privileged lens of twenty-first century scientific insight, however, it is clear this belief system is no longer fit for purpose. The collapse of periodization’s conceptual foundations leaves a void, yet simultaneously creates opportunities to re-evaluate conventional doctrine and to evolve more nuanced and perceptive training planning perspectives. As ever, pockets of innovative coaching practice—both past and present—have already incorporated dimensions of the recommendations noted here into elite training ethos, environments, and systems. Importantly, however, such practices, have been driven primarily by coaching intuition and experience. Such innovations, accordingly, sit outside the boundaries of conventional training theory and remain ignored within the periodization literature. Instead, within that literature, we persist in the cultural conceit that physical training directly and predictably regulates biological adaptation. We portray periodized schemes of empirically described mechanical loads as the epitome of academically validated training planning. We continue to debate the relative worth’s of various periodization models, yet we fail to subject periodization’s foundational precepts to skeptical enquiry.

This rationalization should not be interpreted as an attack on tradition. Previous generations were limited by the informational environments of their time and wisely, we should, of course, respect and learn from those that came before us. We do not, however, honour the past when we cling to convention in the face of disconfirming evidence. The intention here,  accordingly, is simply to highlight that the set of assumptions, presumptions, and rules implicit in periodization theory were formulized under the dictates of a no longer sustainable theoretical reality. In truth, there seems no optimized pre-determinable planning path. There is only the informed exploration of a dynamically changing landscape. An exploration best guided, not by contrived rules and automated decision making, but by critical thinking, examined experience, and the unbiased interpretation of evidence evaluated through a conceptual lens accurately reflecting phenomenological reality.

### Final Comment: Seeking Conceptual Clarity

Given the longevity and publishing productivity of Selye’s career, it would be disingenuous to suggest his perspectives were unchanging or rigidly dogmatic. Importantly, however, his early career breakthroughs were so dominating and so widely publicized that dissent emerged slowly, and somewhat timidly. When disconfirming evidence eventually surpassed credibility thresholds, the field entered what John Mason—the researcher at the forefront of the revolt against stress dogma—described as “*a prolonged period of stalemate and confusion*” [18]. From this confusion, however, greater clarity eventually emerged.

Undoubtedly, Selye’s paradigm contained many partial truths, but its partial validity should not obscure its critical omissions. As we approach the third decade of the twenty-first century, the disconnect between periodization doctrine and both academic insight and progressive coaching practice continues to grow. Resistance to change is not due to a lack of available evidence, such evidence exists; nor a lack of coaching intelligence, which clearly exists. Instead, periodization’s constraining dominance is perpetuated by a path-dependent cultural inertia. An inertia dictating that it is easier to persevere in embedded habits—of thought and practice—than to cut the umbilical cord of convention and re-imagine a new paradigm better fitting contemporary insights.

In moving this field forward, our task is neither to reflexively accept nor automatically reject historical convention. Instead, an awareness of the embedded nature of path-dependent phenomena should encourage us to mindfully scrutinize engrained, often cherished, beliefs so we may better distinguish conveniently simplistic myths from inconveniently complex truths.
